# Supplementary material for: The feasibility of reaching gigatonne scale CO2 storage by mid-century
Source: Nat Commun. 2024 Aug 28;15:6913. doi: 10.1038/s41467-024-51226-8 (PMC11358273; doi:10.1038/s41467-024-51226-8)
Supplement: Supplementary file 1 — Supplementary Information [file 41467_2024_51226_MOESM1_ESM.pdf]

Supplementary Information for the Feasibility of Reaching Gigatonne Scale CO_2 Storage by Mid-century

Yuting Zhang[a*], Christopher Jackson[a], and Samuel Krevor[a]

[a] Department of Earth Science and Engineering, Imperial College London, Exhibition Road, London, SW7 2BX, UK

*Email: yuting.zhang16@imperial.ac.uk

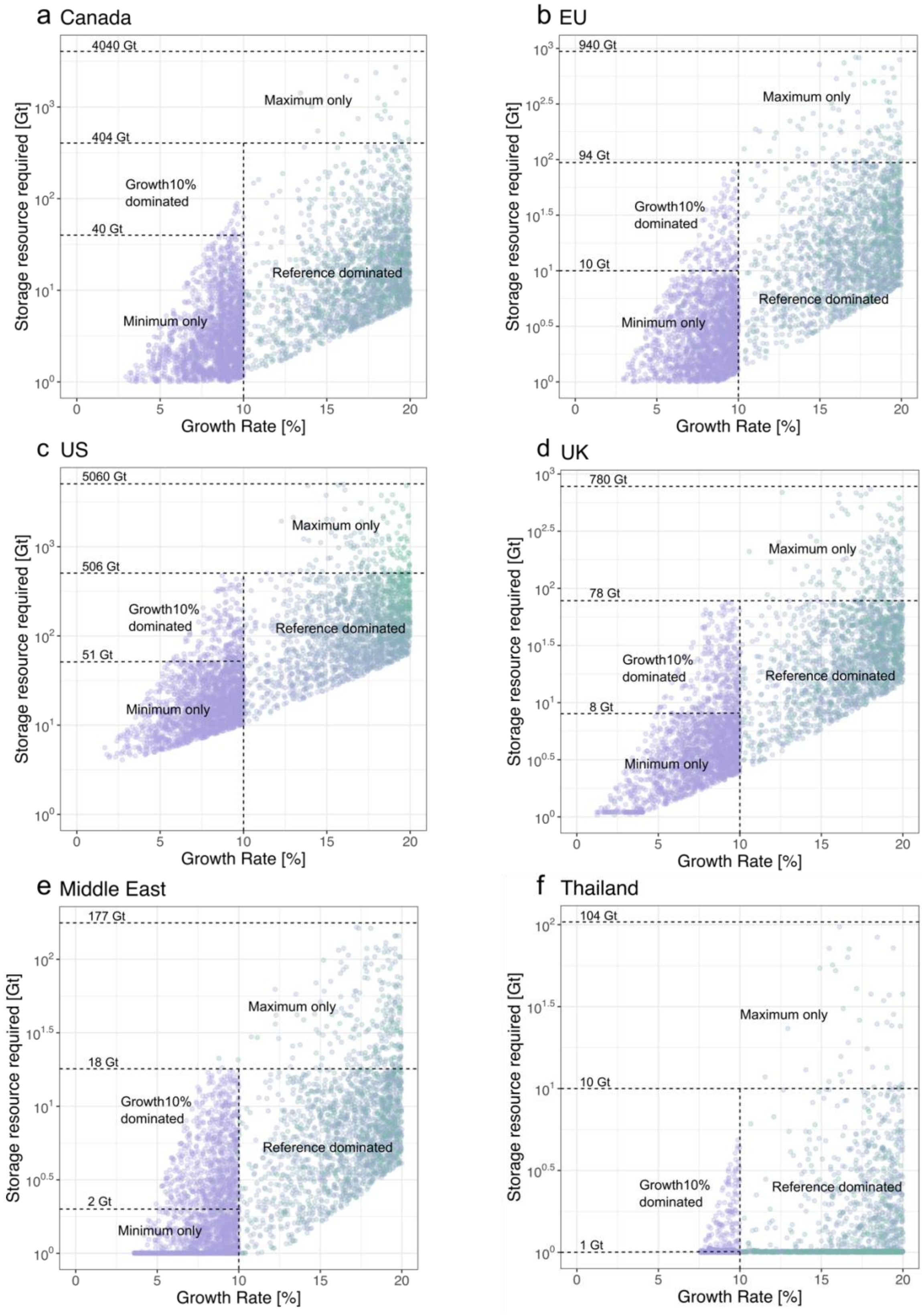

a Canada
4040 Gt
404 Gt
40 Gt
Maximum only
Growth10% dominated
Reference dominated
Minimum only
Storage resource required [Gt]
Growth Rate [%]
b EU
940 Gt
94 Gt
10 Gt
Maximum only
Growth10% dominated
Reference dominated
Minimum only
Storage resource required [Gt]
Growth Rate [%]
c US
5060 Gt
506 Gt
51 Gt
Maximum only
Growth10% dominated
Reference dominated
Minimum only
Storage resource required [Gt]
Growth Rate [%]
d UK
780 Gt
78 Gt
8 Gt
Maximum only
Growth10% dominated
Reference dominated
Minimum only
Storage resource required [Gt]
Growth Rate [%]
e Middle East
177 Gt
18 Gt
2 Gt
Maximum only
Growth10% dominated
Reference dominated
Minimum only
Storage resource required [Gt]
Growth Rate [%]
f Thailand
104 Gt
10 Gt
1 Gt
Maximum only
Growth10% dominated
Reference dominated
Storage resource required [Gt]
Growth Rate [%]

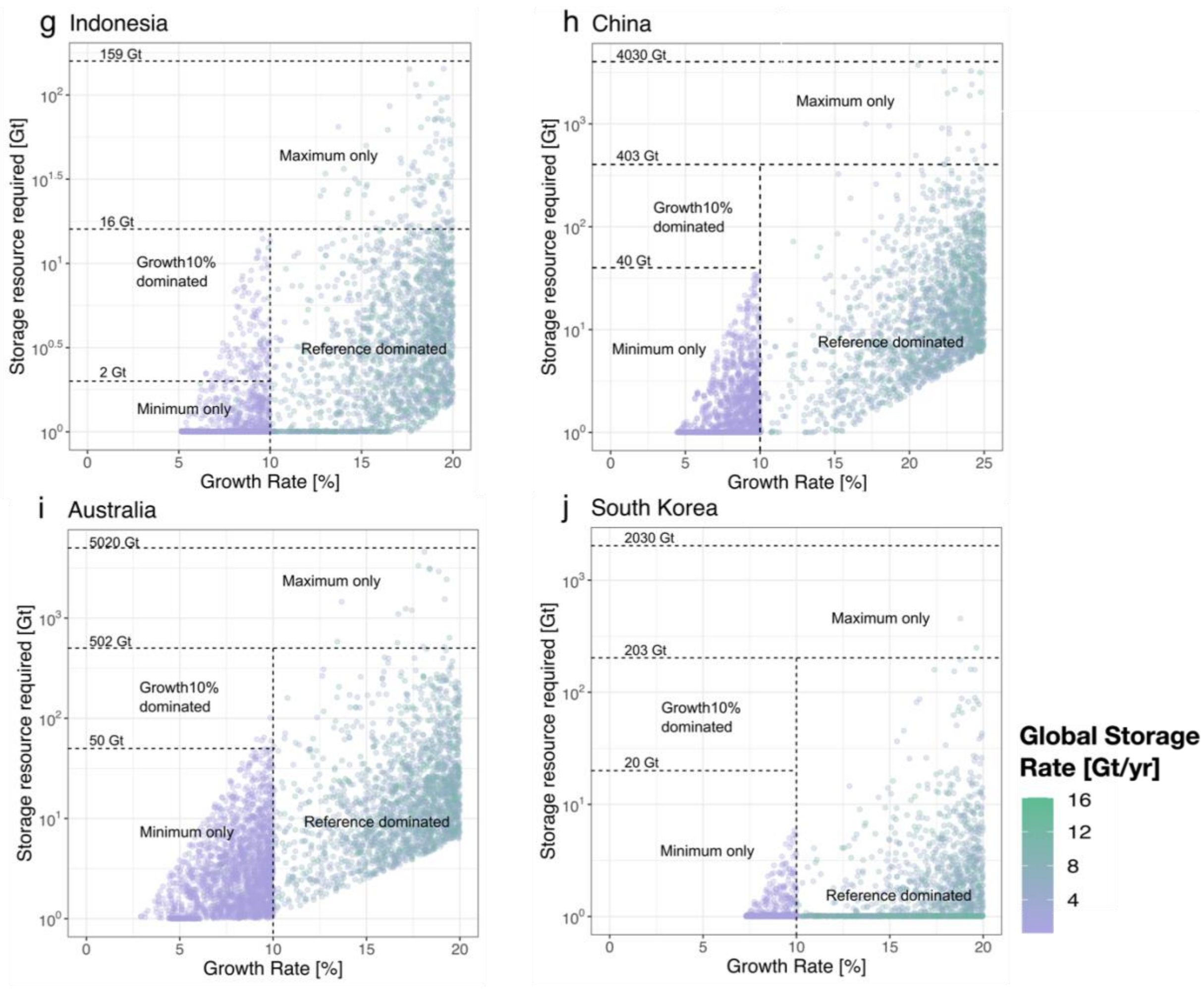

Supplementary Figure 1: **a-j** Base results of geographically resolved CO_2 storage scale up modelled for ten selected storage regions. The points in the graphs each represent a modelled scaleup trajectory, parameterised by the growth rate and storage resource, and within regions of the graph corresponding to the reference, minimum, maximum and growth10% scenarios described in Table 1 of main manuscript. The colour of each marker shows the combined global storage rate where this trajectory is included. The minimum storage resource requirement established as a function of growth rate reflects the parametrisation of peak year which is constrained to post-2050. This consideration reflects a simple representation that the lack of long-term viability of CO_2 storage deployment as a result of a small storage resource base is precluded by the model.
